# Decreased histone deacetylase 4 is associated with human osteoarthritis cartilage degeneration by releasing histone deacetylase 4 inhibition of runt-related transcription factor-2 and increasing osteoarthritis-related genes: a novel mechanism of human osteoarthritis cartilage degeneration

**DOI:** 10.1186/s13075-014-0491-3

**Published:** 2014-11-26

**Authors:** Kun Cao, Lei Wei, Zhiqiang Zhang, Li Guo, Congming Zhang, Yongping Li, Changqi Sun, Xiaojuan Sun, Shaowei Wang, Pengcui Li, Xiaochun Wei

**Affiliations:** Department of Orthopaedics, Second Hospital of Shanxi Medical University, Shanxi Key Laboratory of Bone and Soft Tissue Injury Repair, Taiyuan, China; Department of Orthopaedics and Department of Surgery, Warren Alpert Medical School of Brown University/Rhode Island Hospital (RIH), Providence, Rhode Island USA

## Abstract

**Introduction:**

To investigate if decreased histone deacetylase 4 (HDAC4) is associated with human osteoarthritis (OA) cartilage degeneration by releasing HDAC4 inhibition of runt-related transcription factor-2 (Runx2) resulting in increase of OA cartilage degeneration-related genes.

**Methods:**

The mRNA and protein levels of HDAC4, Runx2, matrix metalloproteinase (MMP)-13, Indian hedgehog (Ihh) and type X collagen were detected by performing real-time PCR (RT-PCR), western blotting and immunohistochemistry on specimens from human OA and normal cartilage. To further explore the mechanism of regulation of Runx2 and OA-related genes by HDAC4, changes in these OA-related genes were further quantified by RT-PCR after overexpression of HDAC4 and knockdown of HDAC4 by siRNA. Runx2 and MMP-13 promoter activities were measured by dual luciferase assays.

**Results:**

The levels of HDAC4 in the cartilage from OA patients and healthy 40- to 60-year-old donors were decreased to 31% and 65% compared with specimens from 20- to 40-year-old healthy donors, respectively (*P* <0.05). Decreased HDAC4 was associated with increased Runx2 and other OA-related genes in human OA cartilage, specifically: MMP-13, Ihh and type X collagen. Exogenous HDAC4 decreased the mRNA levels of Runx2, MMP1, MMP3, MMP-13, type X collagen, Ihh, ADAMTS-4 and -5, and increased the mRNA of type II collagen. In addition, the data also shows that overexpression of HDAC4 not only decreased the expression of interleukin (IL)-1β, Cox2 and iNos and increased the expression of aggrecan, but also partially blocked the effect of IL-1β on expression of catabolic events in human OA chondrocytes. HDAC4 also inhibited Runx2 promoter activity and MMP13 promotor activity in a dose-dependent manner. In contrast, inhibition of HDAC4 by TSA drug had an opposite effect.

**Conclusions:**

Our study is the first to demonstrate that decreased HDAC4 contributes, at least in part, to the pathogenesis of OA cartilage degeneration. Thus, HDAC4 may have chondroprotective properties by inhibiting Runx2 and OA-related genes.

**Electronic supplementary material:**

The online version of this article (doi:10.1186/s13075-014-0491-3) contains supplementary material, which is available to authorized users.

## Introduction

Osteoarthritis (OA) is the most prevalent joint disease, and is characterized by destruction and loss of articular cartilage, leading to chronic pain and dysfunction. Studies have demonstrated that aging, genetics and mechanical factors are associated with the development of OA. However, the molecular mechanisms involved in the pathogenesis and progression of the disease are not completely understood.

Recent studies have demonstrated that changes observed in the behavior of chondrocytes in OA cartilage resemble those undergone by chondrocytes undergoing differentiation during embryogenesis [1-6]. Hypertrophic chondrocytes express matrix metalloproteases (MMPs) and type X collagen, which cause cartilage degradation as part of the endochondral ossification process of growth plate development [7-10]. Chondrocytes residing at the ends of the long bones maintain a stable phenotype and are resistant to the hypertrophic state [11]. However, during OA, chondrocytes lose the stable phenotype and enter hypertrophic differentiation, as demonstrated by increased expression of type X collagen, Ihh, and MMP-13. Cartilage degeneration in OA is therefore similar to cartilage degradation in the growth plate [12,13].

Histone deacetylase 4 (HDAC4) is a member of the class IIa histone deacetylase family. Recent studies show that HADC4 plays an important role in skeletal formation. In the growth plate, HDAC4 prevents chondrocyte hypertrophy by inhibiting the activity of runt-related transcription factor-2 (Runx2), a transcription factor essential for chondrocyte hypertrophy during endochondral bone formation. The HDAC4-null mutant mice display chondrocyte hypertrophy, increased MMP-13, and premature ossification of developing bones due to constitutive expression of Runx2 [14,15]. HDAC4 inhibits MMP-13 through binding to the Runx2 promoter region [15]. Thus, HDAC4 is considered as a central regulator of chondrocyte hypertrophy and skeletogenesis during the growth plate development. However, the role of HDAC4 in OA chondrocytes is unknown. An increase in Runx2 has been reported in OA cartilage and is associated with increases in MMP-13 and type X collagen [16,17]. Moreover, decreased HDAC4 mRNA has been reported in cartilage from OA patients when compared with cartilage from healthy donors [18].

Thus, we put forward the hypothesis that decreased HDAC4 may be associated with human OA cartilage degeneration by releasing HDAC4 inhibition of Runx2 and resulting in increased type X collagen, Ihh, and MMP-13. To test the hypothesis, we first detected and compared the expression of HDAC4, Runx2, and OA-related genes between human OA and normal cartilage. Then we performed a similar comparison after manipulating the expression of HDAC4 in human OA chondrocytes.

## Methods

### Cartilage specimens

Articular cartilage samples were obtained from patients with OA at the time of knee replacement surgery (N = 33, 21 females, 12 males, average age 66.58 ± 7.12 year (mean ± standard deviation (SD)), range 56 to 81 years). Normal samples of articular cartilage were obtained from patients undergoing amputation because of trauma (N = 12, 2 females, 10 males, average age 39.25 ± 13.62, range 21 to 60). The normal samples were divided into two groups based on age: 1) a 20- to 40-year-old group (N = 6, 2 females, 4 males, average age 28.33 ± 7.84, range 20 to 37); and 2) a 40- to 60-year-old group (N = 6, 6 males, average age 50.17 ± 7.78, range 41 to 60). Patients with rheumatoid arthritis and other autoimmune diseases, as well as chondrodysplasias and posttraumatic OA, were excluded from this study. Cartilage specimens from patients with OA were obtained from the more affected compartment (usually medial tibial plateaus). Normal cartilage samples from patients undergoing amputation were also obtained from medial tibial plateaus. The absence of cartilage degeneration in the normal cartilage samples was confirmed using Safranin-O staining. The study was approved by the Institutional Review Board at the Second Hospital of Shanxi Medical University, and informed consent was obtained from each tissue donor.

### Histology

Articular cartilages were fixed in 10% formalin for three days. The specimens were decalcified in EDTA solution, and embedded in a single block of Paraplast X-tra (Thermo Fisher Scientific, Hampton, NH, USA). Serial 6-μm-thick sections were cut by a rotary microtome (Leica RM2125, Leica Microsystems Ltd, Shanghai, China). Safranin-O staining was performed and cartilage damage was quantified using the modified Mankin grading system [19].

### Chondrocyte isolation and primary culture

Chondrocytes were isolated as previously described [20]. Articular cartilage was dissected with a scalpel and subjected to digestion with 2 mg/ml proteinase (Sigma-Aldrich, St Louis, MO, USA) in Hank’s balanced salt solution (HBSS) (Invitrogen, Carlsbad, CA, USA) for 30 min at 37°C with shaking. After digestion and removal of the supernatant, the cartilage pieces were washed with Dulbecco’s modified Eagle’s medium/Ham F-12 (DMEM/F-12) (Gibco BRL, Paisley, UK), and digested with 1 mg/ml Type IA collagenase (Sigma-Aldrich, St Louis, MO, USA) for 6 to 8 h at 37°C with shaking. Enzymatic digestion was stopped by adding DMEM/F-12 containing 10% fetal bovine serum (FBS) (Invitrogen, Carlsbad, CA, USA). Isolated cells were filtered through a 100-μm mesh to remove undigested matrix and the cells were washed three times with DMEM/F-12. Isolated chondrocytes from individual specimens were separately cultured with DMEM/F-12 plus 10% FBS at 37°C under a humidified 5% CO2 atmosphere until reaching confluence. Chondrocyte phenotype were confirmed by immunocytochemistry using anti-type-I and anti-type-II collagen monoclonal antibodies (SC-25974, SC-7764, Santa Cruz Biotechnology, Santa Cruz, CA, USA). These cells are negative for type I staining and positive for type II collagen.

### DNA constructs and transfections

HDAC4-flag and HDAC4-GFP were a generous gift from Dr A. R. Means [21]; pGL3-Runx2-LUC (0.6 kb of rat *RUNX2* promoter) was provided by Dr. G. S. Stein [22]. MMP-13 promoter was a gift provided by Im HJ and Alliston T [23]. The cells were trypsinized and placed in six-well culture plates (Becton Dickinson Labware, Franklin Lakes, NJ, USA) at a density of 1 × 10^6^ cells/plate. The next day, the first passage cells were transfected with HDAC4 small interfering RNA (siRNA) (100 nM), scrambled siRNA control (100 nM) (Cell Signaling Technology, Danvers, MA, USA), or HDAC4-flag or control vector (2.5 μg) using the GeneMute siRNA transfection reagent (SignaGen Laboratories, Gaithersburg, MD, USA). The cells were harvested for real-time PCR or western blot analysis respectively 48 h or 72 h after transfection. To investigate how decreased HDAC4 in articular chondrocytes affects expression of catabolic events in these cells after the treatment with interleukin (IL)-1 beta, the OA chondrocytes were transfected with HDAC4 or control constructs for 48 h, then the cells were stimulated with or without IL-1 beta (5 ng/ul) for 24 h. After IL-1 beta treatment, these cells were harvested for real-time PCR. In order to confirm the transfection efficiency, HDAC4-GFP or siRNA control (fluorescent conjugate) (Cell Signaling Technology, Danvers, MA, USA) were transfected to OA chondrocytes. Forty-eight hours after transfection, live green fluorescent protein (GFP)-positive cells were counted under fluorescence microscopy. Each measurement was made in triplicate.

### Immunohistochemistry staining

Immunohistochemistry was performed to detect the distribution of Runx2、Ihh、type X collagen and MMP-13 in the cartilage specimens. Six-μm paraffin sections were deparaffinized and treated with 3% hydrogen peroxide (Sigma-Aldrich, St Louis, MO, USA) to block intrinsic peroxidase in methanol (Sigma-Aldrich, St Louis, MO, USA) for 30 min. The sections were digested by 5 mg/ml hyaluronic acid (HA) in phosphate-buffered saline (PBS) (Sigma-Aldrich, St Louis, MO, USA) for 20 min. Normal sheep serum (LI-COR Biosciences, Lincoln, NE, USA) was used to prevent nonspecific binding. The sections were incubated with primary antibodies against human Runx2 (CeMines, Inc, Golden, CO, USA), Ihh, MMP-13 (Santa Cruz Technology, Santa Cruz, CA, USA) and type X collagen (EMD, Gibbstown, NJ, USA) respectively at 4°C overnight. The negative control sections were incubated with IgG isotype control (2 mg/ml) (R&D, Minneapolis, MN, USA) in PBS. The second day, the sections were treated sequentially and incubated with biotinylated secondary antibody and streptavidin-peroxidase conjugate (Zymed-Invitrogen, Carlsbad, CA, USA), followed by standardized development in 3′3-diaminobenzidine (DAB). The sections were counterstained with hematoxylin (Zymed-Invitrogen, Carlsbad, CA, USA). Photography was performed with a Nikon E800 microscope (Nikon, Melville, NY, USA).

To detect the expression of HDAC4 in cartilage, 6-μm sections were analyzed by immunofluorescent staining. The sections were incubated with goat primary antibody (sc-5245 Santa Cruz Technology, Santa Cruz, CA, USA), at 4°C overnight. The negative control sections were incubated with isotype control (sc-1196-P, Santa Cruz Technology, Santa Cruz, CA, USA) in PBS. After washing with PBS, affinity-purified tetramethylrhodamine (TRITC)-conjugated donkey anti-goat secondary antibody (1:500) (Jackson ImmunoResearch Laboratories, West Grove, PA, USA) was applied with Hoechst nuclear dye (0.5 mg/ml) (Pierce, Rockford, IL, USA). The sections were washed and mounted in GEL/MOUNT™ (Biomeda Corporation, Foster City, CA, USA). Photography was performed with a Nikon E800 microscope (Nikon, Melville, NY, USA).

To confirm that the OA chondrocytes used in this study were not dedifferentiated, we performed immunohistochemistry with type II collagen and type I collagen staining. 1.5 × 10^5^ cells were seeded on the slide, then fixed in 4% paraformaldehyde for 20 min at 4°C after culture for 48 h. After fixation, cells were permeabilized for 10 min in 0.3% Triton X-100 in PBS and incubated in 3% bovine serum albumin for 1 h. The cells were incubated with primary antibodies against type II collagen and type I collagen overnight at 4°C (SC-25974, SC-7764, Santa Cruz Technology, Santa Cruz, CA, USA). The cells were then incubated with a Texas Red-conjugated secondary antibody (SC-2783, Santa Cruz Technology, Santa Cruz, CA, USA) for 30 min at room temperature, followed by counterstaining with Hoechst 33242 (Pierce, Rockford, IL, USA). Photography was performed with a Nikon E800 microscope (Nikon, Melville, NY, USA).

### Western blotting

Lysis buffer was used to extract total protein from OA cartilage and normal cartilage specimens or from chondrocytes 72 h after transfection with HDAC4 or HDAC4 siRNA. Protein concentrations were determined with the BCA Protein Assay Kit (Pierce, Rockford, IL, USA). Total protein (40 ug) underwent electrophoresis in 10% SDS PAGE gels under reducing conditions. After electrophoresis, proteins were electroblotted onto a PVDF membrane from the gel by a wet blotting apparatus (Thermo Fisher Scientific, Hampton, NH, USA). The membranes were blocked in Tris-buffered saline (TBS) containing 0.1% Tween-20 and 5% dry milk at 4°C overnight. The next day, the membranes were incubated for 6 h at room temperature with primary antibodies against HDAC4, Runx2, Ihh, MMP-13 and Col x. The primary antibodies were diluted 1:500 in TBS containing 0.1% Tween-20 (Sigma-Aldrich, St Louis, MO, USA). Immunoglobulin G (IgG)-conjugated with horseradish peroxidase (HRP) was diluted 1:2000 in TBS containing 0.1% Tween-20 and used as the secondary antibody. The immunoreactive proteins were detected by chemiluminescence using a commercial enhanced chemiluminescence (ECL) kit. Parallel gels were prepared for Coomassie Blue staining to confirm equal loading of total proteins from cartilage samples. The membranes were stripped and probed again with anti-β-actin, to estimate the total protein for equal loading. The densities of bands were quantified using ChemiDoc™ XRS + Software (Bio-Rad, Hercules, CA, USA). The following primary antibodies were used for western blot analysis from Santa Cruz Biotechnology, Santa Cruz, CA, USA. Goat anti-HDAC4 (sc-5245), goat anti-Runx2 (sc-12488), goat anti-collagen 10A1 (sc-323750), goat anti-MMP-13 (sc-31813), goat anti-Ihh (sc-1196), mouse anti-β-actin (sc-130301), rabbit anti-goat IgG-HRP (sc-2768), mouse anti-goat IgG-HRP (sc-2354).

### Real time RT-PCR

Total RNA was isolated from OA cartilage and normal cartilage or from chondrocytes transfected with HDAC4 or HDAC4 siRNA 48 h after the transfection, using Trizol reagent (Invitrogen, Carlsbad, CA, USA). The RNA was reverse-transcribed into first-strand complementary cDNA using the iScript™ cDNA synthesis kit (Bio-Rad, Hercules, CA, USA). The quantification of mRNA was determined using the QuantiTect SYBR Green PCR kit (Qiagen, Valencia, CA, USA) according to the manufacturer’s protocols. Each reaction was performed in triplicate. HADC4 primers were as follows: forward, 5′-GAG AGA CTC ACC CTT CCC G-3′, and reverse, 5′-CCG GTCTGC ACC AAC CAA G-3′. Runx2 primers were as follows: forward, 5′-GGC AGG CAC AGT CTT CCC-3′, and reverse, 5′- GGC CCA GTT CTG AAG CAC C -3′. Ihh primers were as follows: forward, 5′-CAT TGA GAC TTG ACT GGG CAA C-3′, and reverse, 5′-AGA GCA GGC TGA GTT GGG AGTCGC-3′. Type II collagen primers were as follows: forward, 5′-TGA GGG CGC GGT AGA GAC CC-3′, and reverse, 5′-TGC ACA CAG CTG CCA GCC TC-3′. Type X collagen primers were as follows: forward, 5′-GCA CGC AGA ATC CAT CTG AGA ATA-3′, and reverse, 5′-GAC CAG GAG TAC CTT GCT CTC -3′. MMP-1 primers were as follows: forward, 5′-CCA AAT GGG CTT GAA GCT G-3′, and reverse, 5′-GGT ATC CGT GTA GCA CAT TCT GTC--3′. MMP-3 primers were as follows: 5′-TTT CCA GGG ATT GAC TCA AAG A-3′, and reverse, 5′-AAG TGC CCA TAT TGT GCC TTC-3′. MMP-13 primers were as follows: forward, 5′-TGC TGCATT CTC CTT CAG GA-3′, and reverse, 5′-ATG CAT CCA GGG GTC CTGGC-3′. IL-1 beta primers were as follows: forward, 5′-TCG CCA GTG AAA TGA TGG CTT A-3′, and reverse 5′-GTC CAT GGC CAC AAC AAC TGA-3′. iNos primers were as follows: forward, 5′-CCT TAC GAG GCG AAG AAG GAC AG-3′, and reverse, 5′-CAG TTT GAG AGA GGA GGC TCC G-3′. Cox2 primers were as follows: forward, 5′-GAG AGATGTATC CTC CCA CAG TCA-3′, and reverse, 5′- GAC CAG GCA CCA GAC CAA AG-3′. ADAMTS-4 primers were as follows: forward, 5′-GAC ACG CTG GGT ATG GCT GA-3′, and reverse, 5′-ACT GAT GCA TGG CTT GGA GTT G-3′. ADAMTS-5 primers were as follows: forward, 5′-TAT GAC AAG TGC GGA GTA TG -3′ and reverse, 5′-TTC AGG GCT AAA TAG GCA GT-3′. Aggrecan primers were as follows: forward, 5′- CAT TCA CCA GTG AGG ACC TCG T-3′ and forward, 5′-TCA CAC TGC TCA TAG CCT GCT TC-3′. Amplification conditions were as follows: 2 min of preincubation at 50°C, 10 min at 95°C for enzyme activation, and 40 cycles at 95°C denaturation for 10 s, 55°C annealing for 30 s, and 72°C extension for 30 s. 18S was used as an internal control gene to normalize the mRNA levels. The cycle threshold (Ct) values for 18 s RNA and that of target genes were measured and calculated by computer software (IQ50, Bio-Rad Laboratories, Hercules, CA, USA). Relative transcription levels were calculated as x = 2^-ΔΔCT^, was used for the calculation of fold amplification [24].

### Reporter gene assays

Prehypertrophic chondrocytes were isolated from the cephalic part of the sternum of a 17-day-old embryonic chick as previously described [25,26]. Human chondrocyte cell line (C28/12) and chicken chondrocytes were transiently transfected with 0.5 μg of Runx2 or MMP-13 promoter luciferase reporter plasmid using 5 μL/well of lipofectamine 2000 (Invitrogen, Carlsbad, CA, USA) in 12-well plates. Co-transfections with promoters were performed with empty vector or different concentrations of expression constructs of HDAC4 as indicated. In some case, the cells transfected with promoters were treated with different concentrations of HDAC inhibitor trichostatin A (TSA) as indicated. Cells were harvested in 1 × reporter lysis buffer 48 h after transfection. Luciferase activity of Runx2 was read in a luminometer using the dual luciferase reporter assay and normalized to Renilla activity (Dual-Luciferase Reporter Assay, Promega, Madison, WI, USA). Each measurement was made in triplicate.

### Statistical analysis

Analysis of variance (ANOVA) was used to compare the significant differences among the concentrations of HDAC4 in normal age-matched control and OA cartilage specimens. Two-tailed paired *t* tests were used to compare statistically significant differences of the protein (MMP-13, Ihh, type X collagen) in OA and control cartilage specimens. Student’s *t* test was used to compare mRNA levels in cartilage or chondrocytes. ANOVA was used to compare significant differences among the effects on promoter activity of Runx2 or MMP13 with different concentrations of HDAC4 constructs or TSA. ANOVA was used to compare significant differences among the effects of HDAC4 on catabolic markers in IL-beta-treated chondrocytes. *P* <0.05 was considered significant.

## Results

### Decrease of HDAC4 protein found by western blotting in human aging and OA cartilage

**Figure 1:**
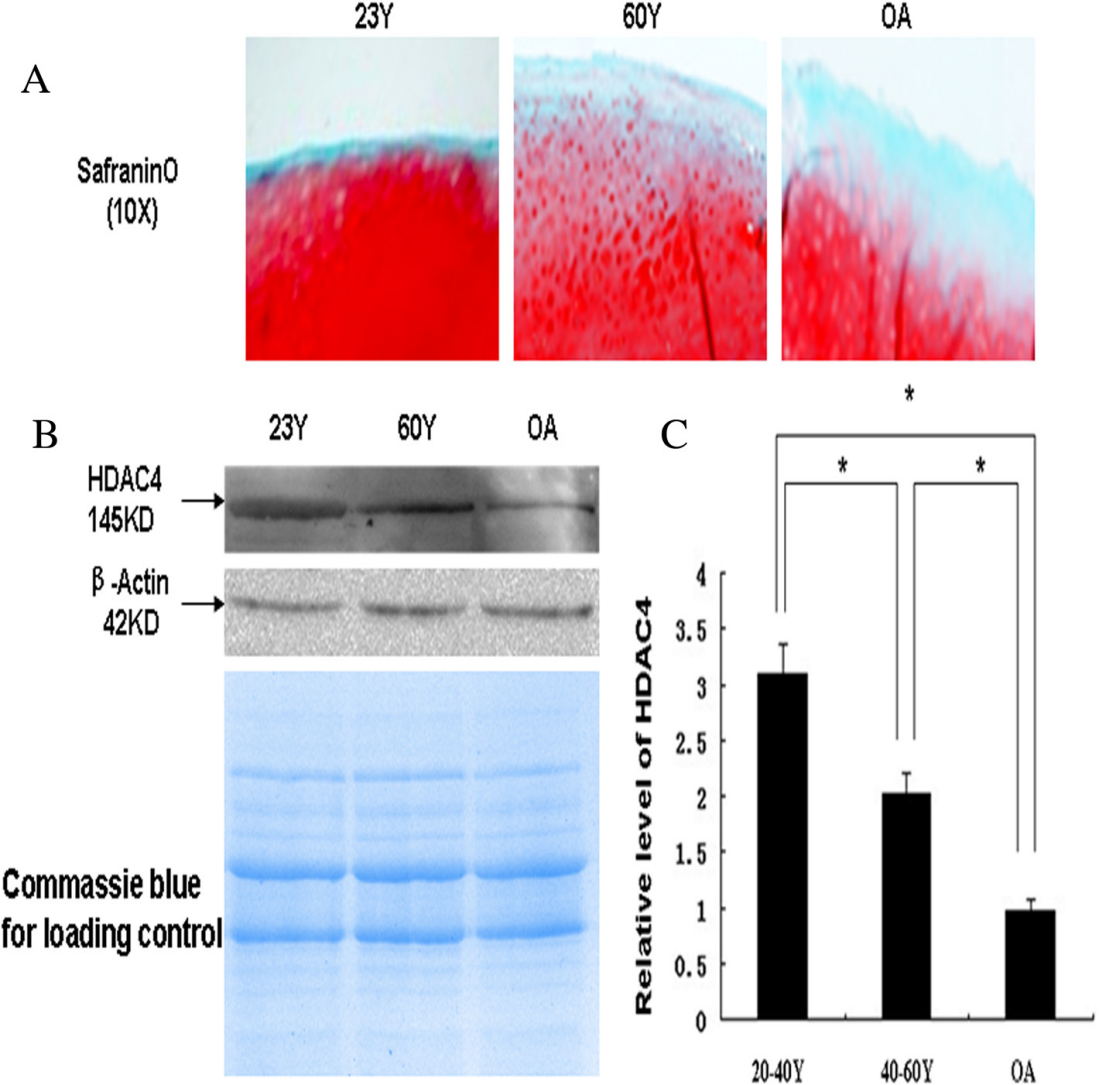
**Decrease of HDAC4 protein determined by western blotting in human aging and OA cartilage specimens. (A)** Safranin O staining was performed to show degeneration changes in the cartilage from a 61-year-old OA patient undergoing total knee arthroplasty, and the lack thereof in normal cartilage from a 60-year-old male patient and 23-year-old male patient undergoing amputation because of trauma. **(B)** Western blotting showed decreased expression of HDAC4 in human aging and OA cartilage. β-actin and Coomassie Blue staining was used to confirm equal loading of total proteins from cartilage samples. **(C)** Semi-quality by densitometry showed that the mean concentration of HDAC4 was 31% and 65% in the cartilage of OA patients (n = 6) and the 40 to 60 age group (n = 6), respectively, compared with the cartilage of the 20 to 40 age group (n = 6). ^*^ *P* represents that the difference was statistically significant (*P* <0.05). HDAC4, histone deacetylase 4; OA, osteoarthritis.

First, we analyzed expression of HDAC4 in cartilage samples from the knee joints of OA patients and compared it to that in normal cartilage samples from 20- to 40-year-olds and 40- to 60-year-olds. Safranin O staining demonstrated degeneration in OA cartilage, and the lack thereof in normal cartilage (Figure 1A). Western blotting analysis showed that expression of HDAC4 were decreased in the 40- to 60-year-olds and further decreased in the OA specimens, compared with the specimens obtained from the 20- to 40-year-olds (Figure 1B). β-actin and Coomassie Blue staining confirmed equal loading of total proteins from cartilage samples (Figure 1B). Semi-quantification of western blots showed that, compared with cartilage specimens from the 20- to 40-year-old group, those from the OA and 40- to 60-year-old group had 31% and 65% of the concentration of HDAC4, respectively. (*P* <0.05) (Figure 1C).

### Increased expression of RUNX2 was associated with decreased HDAC4 in OA cartilage

**Figure 2:**
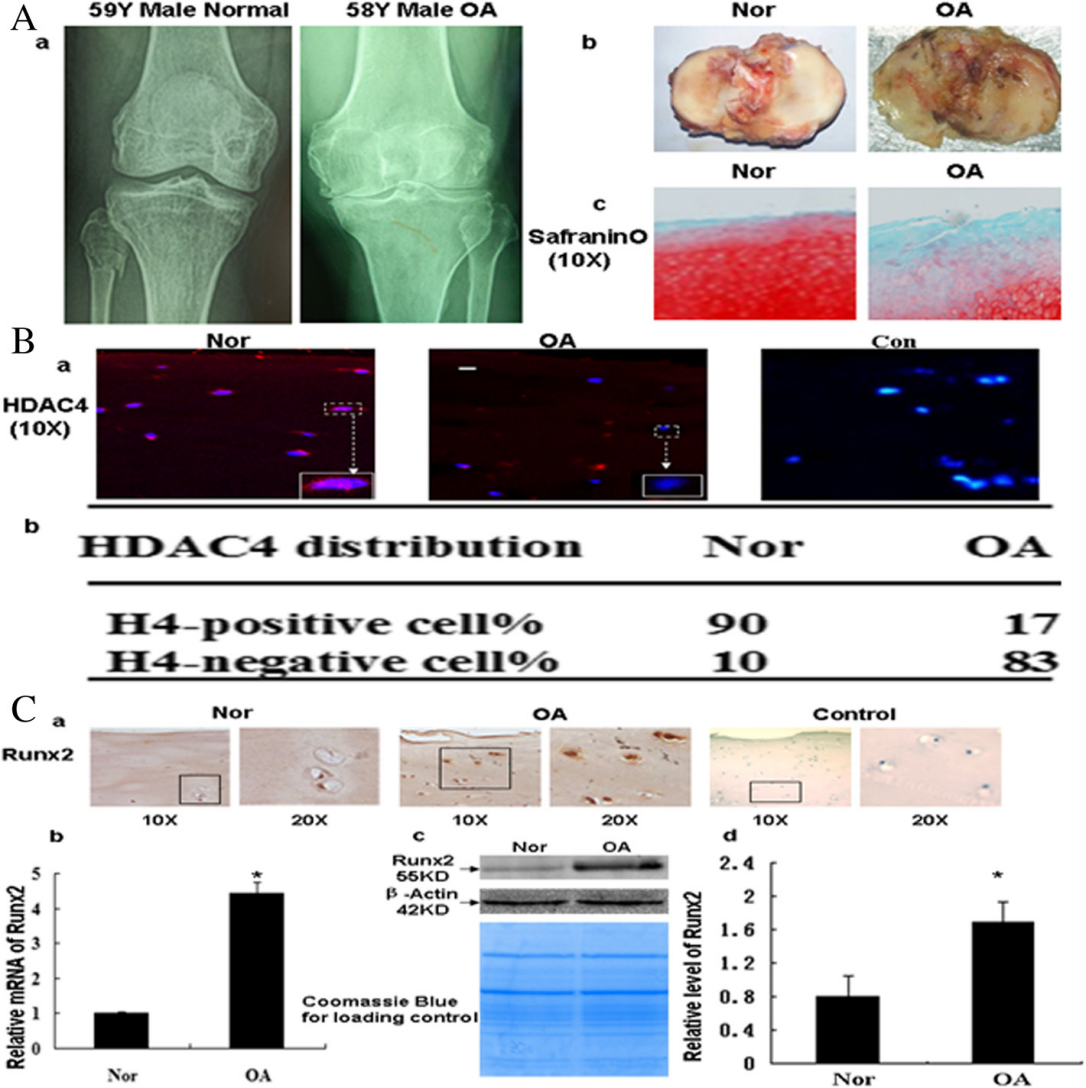
**Increased expression of RUNX2 associated with decreased HDAC4 in OA cartilage. (A-a)** X-ray film showed no radiographic changes in the normal controls, whereas there was joint space narrowing and cartilage damage in the OA patients. **(A-b)** The tibial plateaus from normal control and OA patients were shown **(A-c)**. Safranin O staining was performed to show degeneration in the cartilage from OA patients and the lack thereof in normal control. **(B-a, B-b)** Immunofluorescent histochemistry was used to show HDAC4 staining (blue: nucleus; red: HDAC4). HDAC4-positive cells (red) were significantly less in OA cartilage than the normal control. The upper boxed region indicates the magnified area shown at the boxed region below. HDAC4-positive cells were decreased to 17% of total cells in OA cartilage compared with 90% in normal control. **(C-a)** Immunohistochemistry was performed to show expression of Runx2 in OA cartilage and normal control. The boxed region indicates the magnified area shown at the right side. **(C-b)** The mRNA levels of Runx2 in OA cartilages (n = 3) and normal controls (n = 6) were tested by real-time PCR. (^*^ *P* <0.05) **(C-c,C-d)** Western blotting showed expression of Runx2 increased in OA samples compared with the normal control. Semi-quality by densitometry showed the mean concentration of Runx2 in the cartilage of OA patient (n = 3) compared with the cartilage of normal controls (40- to 60-year- olds) (n = 3) (^*^ *P* <0.05). HDAC4, histone deacetylase 4; OA, osteoarthritis; Runx2, runt-related transcription factor-2.

To evaluate the potential relationship between HDAC4 and Runx2 as dysregulated pathways in OA cartilage, we analyzed expression of HDAC4 and Runx2 in cartilage from OA patients and age-matched normal cartilage specimens from the 40- to 60-year-old group (herein after called ‘normal cartilage’). Immunofluorescent staining for HDAC4 was significantly lower in OA cartilage specimens compared with normal cartilage (Figure 2B). HDAC4-positive cells were 17% of total cells in OA cartilage compared with 90% in normal cartilage (*P* <0.05) (Figure 2B). Immunohistochemistry staining showed increased Runx2 in OA cartilage compared with normal cartilage (Figure 2C-a), and real-time PCR and western blotting yielded similar findings (Figure 2C-b,C-c,C-d). Runx2 gene expression was increased 4.4-fold in OA cartilage compared with the normal controls. (*P* <0.05) (Figure 2C-b). Quantification of western blots showed that the concentration of Runx2 in the cartilages of OA patients was increased 212% compared with the normal cartilages (*P* <0.05) (Figure 2C-d).

### Increased MMP-13, Ihh and type X collagen expression in OA cartilage

**Figure 3:**
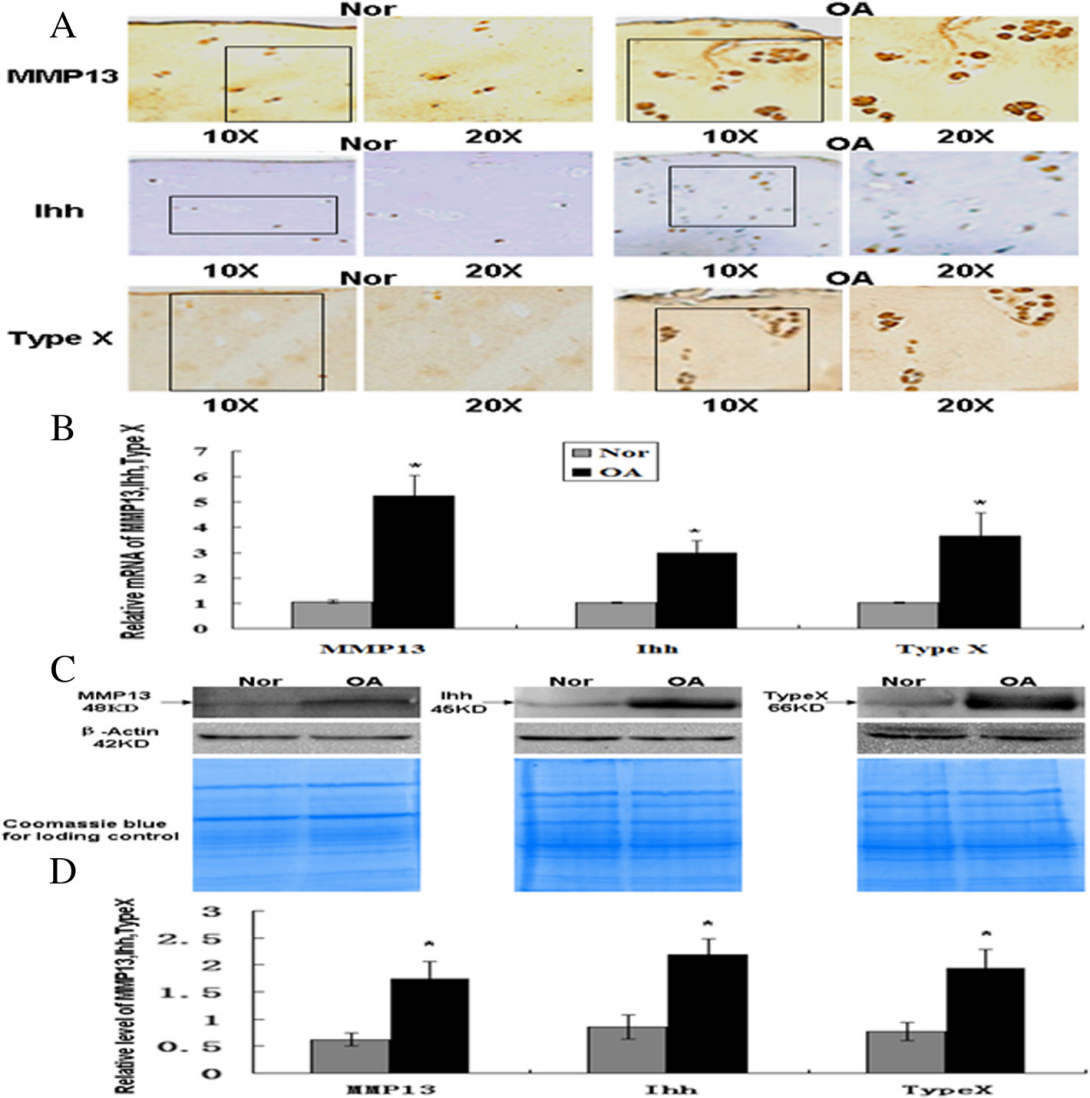
**Increased MMP-13, Ihh and type X collagen expression in OA cartilage. (A)** Immunohistochemistry was performed to detect MMP-13, Ihh and type X collagen. The boxed regions indicate the magnified area shown at the right side. **(B)** Real-time PCR indicates that the level of MMP-13, Ihh and type X collagen increased in OA cartilages (n = 3) compared with normal cartilage (n = 3) (^*^ *P* <0.05). **(C, D)** Western blotting showed expression of MMP-13, Ihh and type X collagen increased in OA samples compared with normal controls. Semi-quality by densitometry showed the mean concentration of MMP-13, Ihh and type X collagen in the cartilage of OA patients (n = 3) compared with the cartilage of normal controls (40- to 60-year-olds) (n = 3) (^*^ *P* <0.05). MMP, matrix metalloprotease; OA, osteoarthritis; SD, standard deviation.

Strong staining for MMP-13, Ihh and type X collagen was observed in OA cartilage compared with normal cartilage (Figure 3A). Real-time PCR results indicated that the level of MMP-13, Ihh and type X collagen increased 5.24-, 2.98- and 3.65-fold, respectively, in OA cartilages compared with normal cartilage (*P* <0.05) (Figure 3B). Similar findings were made by western blotting. (Figure 3C). Semi-quantification of western blots showed that the concentration of MMP-13, Ihh and type X collagen was increased 278%, 257% and 251% in cartilage from OA patients compared with those from normal controls, respectively (*P* <0.05) (Figure 3C,D).

### HDAC4 inhibits Runx2 and MMP-13 expression in human OA chondrocytes

**Figure 4:**
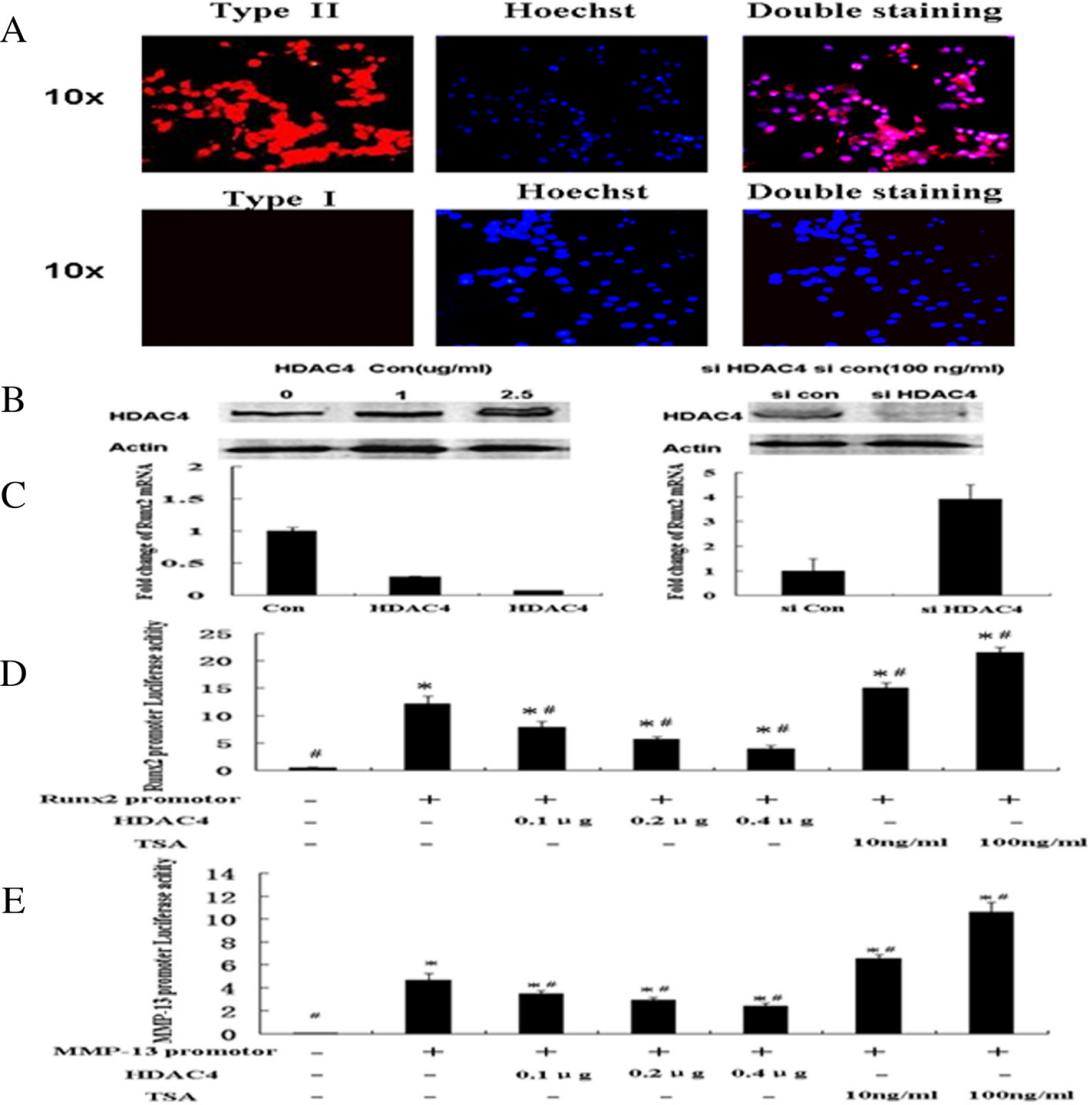
**HDAC4 inhibits Runx2 and MMP-13 expression in human OA chondrocytes. (A)** Human OA chondrocyte phenotype was confirmed by immunocytochemistry. The cells were positive for type II, but not for type I collagen. **(B)** Transfection efficiency of HDAC4 in OA chondrocytes was validated by western blotting. **(C)** Real-time PCR results indicated that overexpression of HDAC4 downregulated Runx2. The experiments were performed in triplicate. Data are presented as means ± SD. **(D)** HDAC4 inhibited Runx2 promoter activity in the human chondrocyte cell line (C28/12) in a dose-dependent manner. Conversely, inhibition of HDAC by TSA drug enhanced Runx2 promoter activity in a dose-dependent manner. ^*^ = *P* <0.05 versus control OA culture; # = *P* <0.05 versus the group of Runx2 promoter transfection. **(E)** HDAC4 inhibited MMP-13 promoter activity in the human chondrocyte cell line (C28/12) in a dose-dependent manner. Conversely, inhibition of HDAC by TSA drug enhanced MMP-13 promoter activity in a dose-dependent manner. All transfections were performed in triplicate. ^*^ = *P* <0.05 versus control OA culture; # = *P* <0.05 versus the group of Runx2 promoter transfection. Data are presented as means ± SD. Values represent the mean ± SD from three independent experiments. HDAC4, histone deacetylase 4; MMP, matrix metalloprotease; OA, osteoarthritis; Runx2, runt-related transcription factor-2; SD, standard deviation; TSA, trichostatin A.

Primary chondrocyte phenotype was confirmed by immunocytochemistry, with negative type I staining and positive type II collagen (Figure 4A). To confirm the transfection efficiency, HDAC4-GFP or siRNA control (fluorescent conjugate) were transfected to OA chondrocytes. Our data demonstrated that the average transfection efficiency of GFP-HDAC4 or siRNA control was around 79.1% (72.6% to 87.6%) or 88.2% (85.4% to 91.2%), respectively (Figure S1 in Additional file 1). To test whether decreased HDAC4 was associated with increased Runx2 in OA cartilages, OA chondrocytes were transfected with different concentrations of HDAC4 (1 μg or 2.5 μg) or HDAC4 siRNA (100nM) against HDAC4. Real-time PCR demonstrated that HDAC4 inhibited Runx2 mRNA in a dose-dependent manner. The mRNA levels of Runx2 in OA chondrocytes transfected with low and high concentration of HDAC4 decreased to 29% and 7% respectively, of those in the control vector (Figure 4C). Increased HDAC4 protein was confirmed by western blotting (Figure 4B). Conversely, increased mRNA of Runx2 was observed in the cells transfected with HDAC4 siRNA. Western blotting showed that HDAC4 siRNA effectively knock down the expression of HDAC4, whereas Runx2 mRNA increased 3.9-fold (Figure 4B, C). To further explore the mechanism of the regulation of Runx2 and MMP-13 by HDAC4, human chondrocyte cell line (C28/12) and chicken chondrocytes (data not shown) transfected with Runx2 or MMP-13 reporter constructs were treated or co-transfected with different concentrations of TSA (10 ng/ml or 100 ng/ml) or HDAC4 (0.1 μg, 0.2 μg or 0.4 μg) respectively. Dual luciferase assay was performed 48 h after transfection. Runx2 or MMP-13 promoter activity was repressed in a dose-dependent manner by HDAC4. Conversely, inhibition of HDAC by TSA increases Runx2 or MMP-13 promoter activity in a dose-dependent manner (*P* <0.05) (Figure 4D, E).

### HDAC4 downregulates OA-related gene expressions in human OA chondrocytes

**Figure 5:**
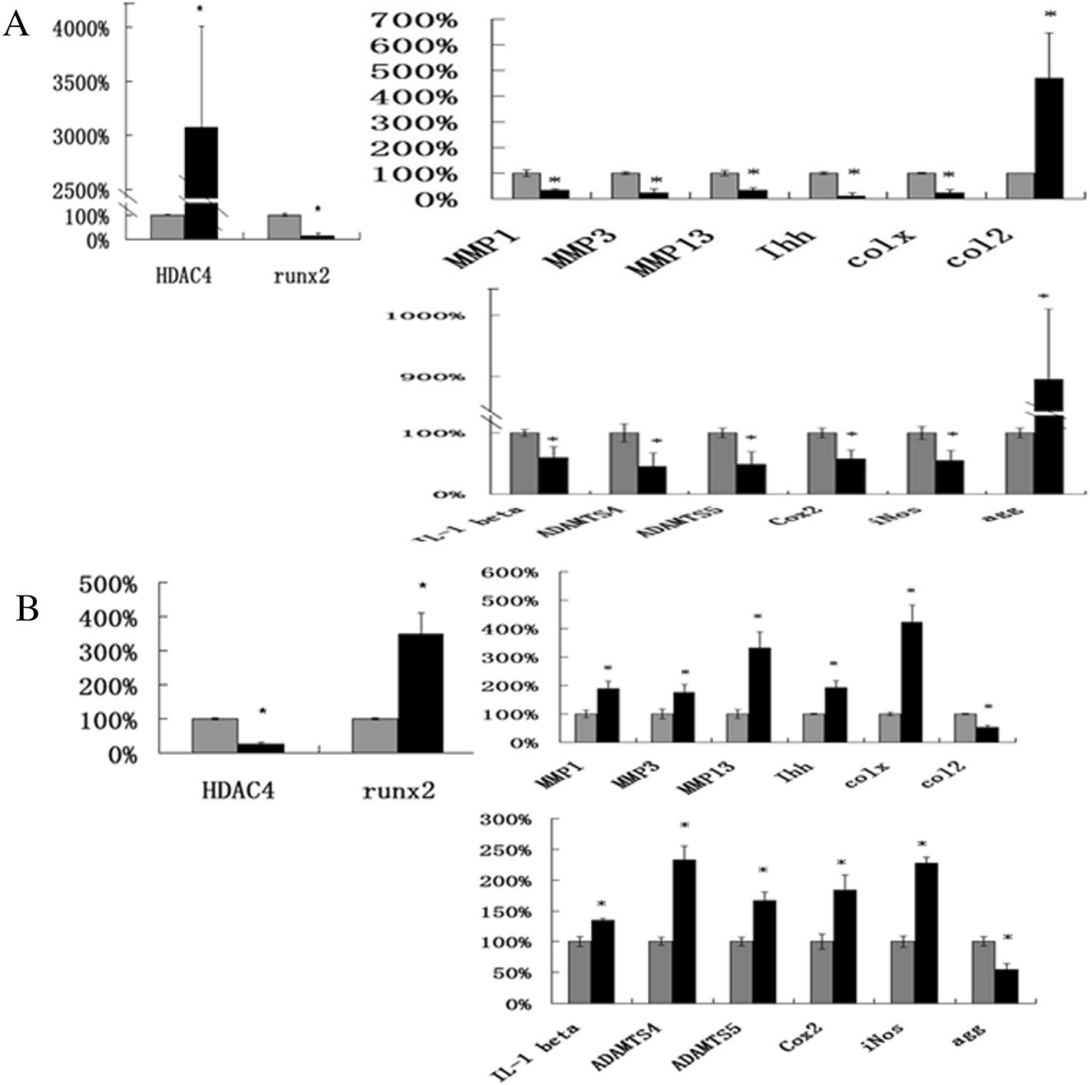
**HDAC4 downregulates OA-related gene expressions in human OA chondrocytes.** Human OA chondrocytes were transfected with HDAC4-flag or HDAC4 siRNA. All transfections were performed in triplicate. Data are presented as means ± SD. ^*^ *P* <0.05. **(A)** The mRNA of HDAC4, Runx2, MMP1, MMP3, MMP-13, type X collagen, Ihh, type II collagen, IL-1 beta, Cox2, iNos, ADAMTS-4 and -5 was tested by real-time PCR after HDAC4 or control vector transfection. **(B)**The mRNA of HDAC4, Runx2, MMP1, MMP3, MMP-13, type X collagen, Ihh, type II collagen, IL-1 beta, Cox2, iNos, ADAMTS-4 and -5 was tested by real-time PCR after HDAC4 siRNA or scrambled siRNA control transfection. HDAC4, histone deacetylase 4; MMP, matrix metalloprotease; OA, osteoarthritis; Runx2, runt-related transcription factor-2; SD, standard deviation; siRNA, small interfering RNA; TSA, trichostatin A.

Our data showed that overexpression of HDAC4 decreased the mRNA levels of Ihh, type X collagen, MMP1, MMP3, MMP-13, IL-1 beta, Cox2, iNos, ADAMTS-4 and -5 in OA chondrocytes to 11%, 23%, 33.8%, 23.7%, 33%, 60%, 57.7%, 54.7%, 45.1% and 48.9%, respectively, of those in controls (Figure 5A). In addition, the mRNA levels of type II collagen and aggrecan significantly increased 4.7- and 9.0-fold after HDAC4 transfection (Figure 5A) (*P* <0.05). In contrast, knockdown HDAC4 by HDAC4 siRNA showed an opposite effect (Figure 5B) (*P* <0.05). The transfection efficiencies of HDAC4 and HDAC4 siRNA were validated by real-time PCR (Figure 5A, B).

### HDAC4 partially blocks the effect of IL-1 beta on expression of catabolic events in human OA chondrocytes

**Figure 6:**
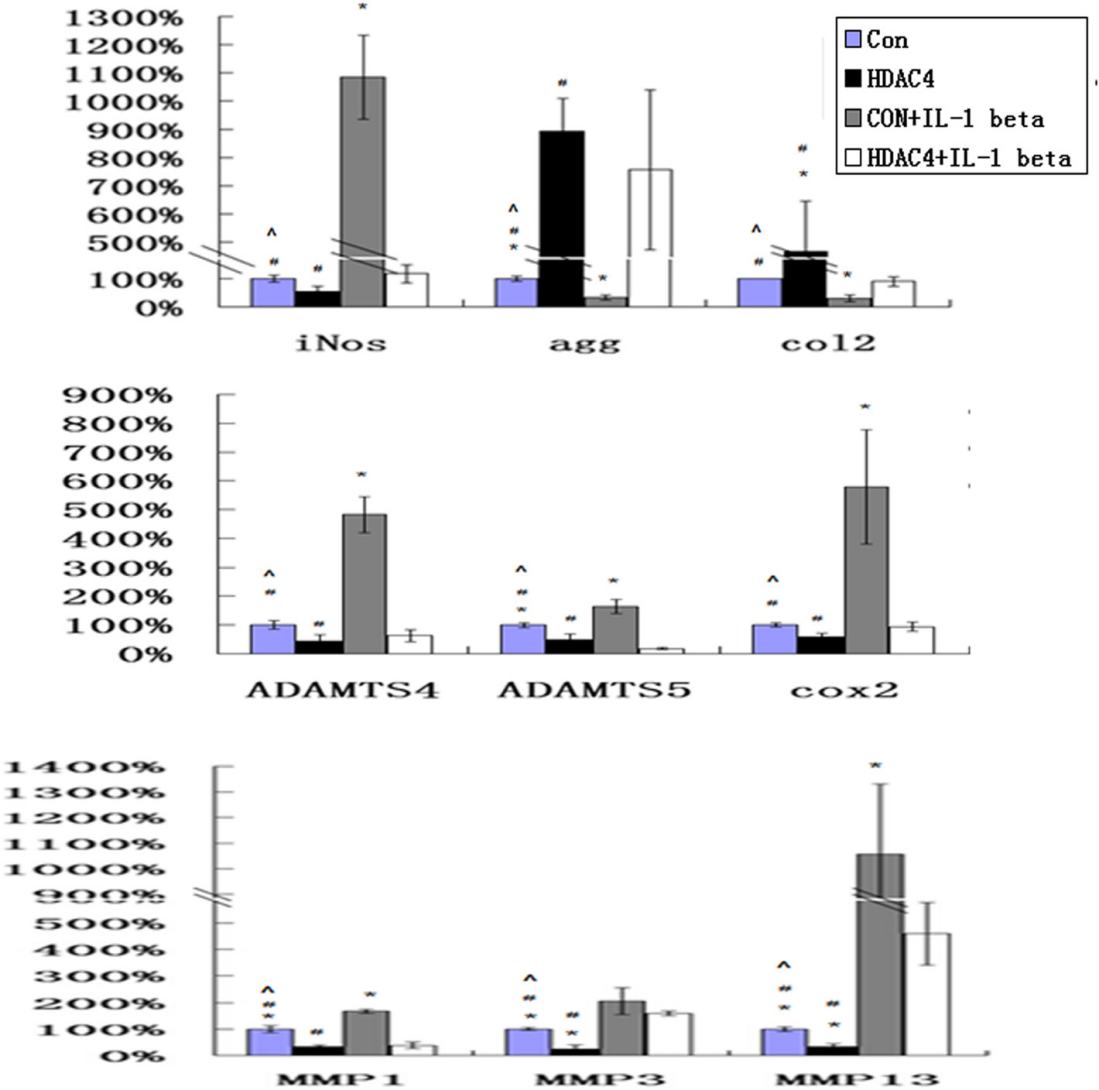
**HDAC4 partially blocks the effect of IL-1 beta on expression of catabolic events in human OA chondrocytes.** Human OA chondrocytes were transfected with HDAC4 or control constructs for 48 h, then the cells were stimulated with or without IL-1 beta (5 ng/ ul) for 24 h. All transfections were performed in triplicate. ^*^ = *P* <0.05 versus the group of HDAC4 + IL-1 beta, # = *P* <0.05 versus the group of control vercor, ^ = *P* <0.05 versus the group of control vector + IL-1 beta. Real-time PCR showed that IL-1 beta significantly increased the mRNA levels of MMP-1, MMP3, MMP-13, iNOS, Cox2, ADAMTS-4 and -5 in the OA chondrocytes, respectively compared to those untreated controls. In addition, the mRNA levels of aggrecan, and type II collagen significantly decreased after stimulation with IL-1 beta (*P* <0.05). Overexpression of HDAC4 partly blocked the upregulation of MMP-1, MMP3, MMP-13, iNOS, Cox2, ADAMTS-4, and -5 induced by IL-1 beta. In the meanwhile, the mRNA levels of aggrecan, and type II collagen significantly increased in those cells transfected by HDAC4 (*P* <0.05) (n = 3). HDAC4, histone deacetylase 4; IL, interleukin; MMP, matrix metalloprotease; OA, osteoarthritis; Runx2, runt-related transcription factor-2.

Real-time PCR showed that IL-1 beta significantly increased the mRNA levels of MMP-1, MMP3, MMP-13, iNOS, Cox2, ADAMTS-4 and -5 in the OA chondrocytes, respectively compared to those untreated controls. In addition, the mRNA levels of aggrecan, and type II collagen significantly decreased after stimulation with IL-1 beta. Overexpression of HDAC4 partly blocked the upregulation of MMP-1, MMP3, MMP-13, iNOS, Cox2, ADAMTS-4, and -5 induced by IL-1 beta. In the meanwhile, the mRNA levels of aggrecan, and type II collagen significantly increased in those cells transfected by HDAC4 (Figure 6) (*P* <0.05).

## Discussion

Studies have demonstrated that the class II HDACs are involved in cellular development and differentiation, and expressed in a tissue-specific pattern. HDAC4 is expressed in brain, muscle and cartilage [27,28]. In the growth plate, HDAC4 is a negative regulator of chondrocyte hypertrophy by binding to and inhibiting Runx2 - a critical transcriptional factor for chondrocyte hypertrophy [14,26]. However, the role of HDAC4 during OA cartilage degeneration is unknown. In this study, for the first time, we found that expression of HDAC4 protein decreases with aging in human cartilage, and furthermore in human OA cartilage (Figure 1).

In addition to decreased HDAC4, we found that expression of Runx2, Ihh, MMP-13 and type X collagen is increased in OA cartilage with respect to normal controls (Figures 2 and 3). This result is also consistent with previous reports [20,29-32]. Runx2 is a critical regulator for chondrocyte hypertrophy and contributes to the progression of OA. Previous studies have reported that Runx2 is activated in OA cartilage and responsible for the increase of MMP-13 and type X collagen in a mouse OA model and in human OA chondrocytes [8,10,17,33]. Type X collagen and MMP-13 are markers for chondrocyte hypertrophy, and MMP-13 is a primary player in OA cartilage damage [32,34-36]. In an *in vivo* study, the heterozygous Runx2-deficient mice showed decreased cartilage damage and reduced type X collagen and MMP-13 expression compared with wild-type mice [10]. These findings suggest that increased Runx2 in OA cartilage may trigger the expression of a series of genes related to OA cartilage damage. However, it is unknown how Runx2 is regulated in OA cartilage. Taken together with the dysregulation of the genes in OA cartilage shown in our study (Figures 2 and 3), it is likely that decreased HDAC4 is at least partially responsible for the increase of Runx2 and OA-related genes in OA cartilage. We hypothesize that decreased HDAC4 in OA cartilage will release the HDAC4 inhibition of Runx2, thus resulting in the increase of Runx2 activity. Thus, upregulation of Runx2 activates a series of OA-related genes and results in cartilage degeneration.

To test whether decreased HDAC4 makes a contribution to increased Runx2 and other OA-related genes in human OA cartilage, OA chondrocytes were transfected with either HDAC4 expression construct or siRNA against HDAC4. Our data showed that either the mRNA level of Runx2 were repressed after overexpression of HDAC4, and the opposite effect was observed when HDAC4 was knocked down by siRNA (Figures 4 and 5). Moreover, we also demonstrated that HDAC4 inhibited Runx2 and MMP-13 promoter activities in a dose-dependent manner (Figure 4D, E). In contrast, inhibition of HDAC4 by TSA increased Runx2 and MMP-13 promoter activities. HDAC4 and Runx2 are known as important regulators of chondrocyte hypertrophy during growth plate development. It is reported that HDAC4 repressed chondrocyte hypertrophy by binding to and inhibiting Runx2 transcriptional activity in the chondrocytes of an embryonic chick [26]. HDAC4-null mice display ectopic and premature mineralization of endochondral bones, mimicking the Runx2 gain of function mice [14]. Consistent with this finding, Runx2, Ihh and MMP-13 are greatly increased in HDAC4−/− mice [14,15]. It is also reported that HDAC4 inhibited MMP-13 through Runx2 and HDAC4 and 5 were required for transforming growth factor-β-mediated repression on Runx2 function to inhibit osteoblast differentiation [15,37]. A mechanism of HDAC4 repression on Runx2 has been proposed in which HDAC4 interacts with Runx2 protein to deacetylate Runx2 [38]. Our results indicate the mechanism of HDAC4 acting on Runx2 and MMP-13 in OA chondrocytes is similar to that found in the growth plate and osteoblasts.

In this study, the reduction of endogenous HDAC4 contributes to the increase of Runx2, type X collagen, and MMP-13 in OA chondrocytes. We found that the overexpression of HDAC4 decreased the mRNA level of MMP1, MMP3, MMP-13, type X collagen, Ihh ADAMTS-4 and -5, and increased the mRNA of type II collagen. HDAC4 inhibits chondrocyte hypertrophy by binding to and inhibiting Runx2 and MMP-13 [14,26]. Runx2 is a well-known transcriptional factor that induces MMP-13, Ihh and ADAMTS-5 production [14,15,39]. Increased Ihh was associated with chondrocyte hypertrophy and upregulation of MMP1, MMP3, and MMP-13 in a mouse OA model and human OA cartilage [20,40]. Thus, a mechanism of HDAC4 repression on Runx2 is involved in this phenomenon. Our data also shows that overexpression of HDAC4 not only decreased the expression of IL-1 beta, Cox2 and iNos and increased the expression of aggrecan, but also partially blocked the effect of IL-1 beta on expression of catabolic events in human OA chondrocytes. IL-1 beta plays a critical role in the cartilage degeneration [41-43]. However, the mechanism by which HDAC4 regulates IL-1 beta is unclear. Future studies are necessary to investigate the mechanism of HDAC4 action on IL-1 beta. These results suggest that upregulation of HDAC4 may have a chondroprotective effect in patients with early-stage disease.

## Conclusions

In summary, our novel findings indicate, for the first time, that decreased HDAC4 is at least partially responsible for the increase of Runx2 and OA-related genes in human OA cartilage. This finding will facilitate our understanding of the pathogenesis of degenerative cartilage. Thus, upregulation of HDAC4 may have a chondroprotective effect in OA therapy.

## Additional file

Additional file 1: Figure S1. The transfection efficiency of GFP-HDAC4 or siRNA control. **(A)** Efficiency of GFP-HDAC4 transfection was 79.1% (72.6% to 87.6%). **(B)** Efficiency of siRNA control transfection was 88.2% (85.4% to 91.2%). The percentage of cells that are GFP-positive was detected using fluorescence microscopy. Approximately 300 cells from three independent experiments were scored.

## Abbreviations

ANOVA: analysis of variance
DMEM: Dulbecco’s modified Eagle’s medium
FBS: fetal bovine serum
GEP: green fluorescent protein
HDAC4: histone deacetylase 4
IgG: immunoglobulin G
IL: interleukin
MMPs: matrix metalloproteases
OA: osteoarthritis
PBS: phosphate-buffered saline
Runx2: runt-related transcription factor-2
SD: standard deviation
siRNA: small interfering RNA
TBS: Tris-buffered saline
TSA: trichostatin A
