# Supplementary figures and images for: Decreased histone deacetylase 4 is associated with human osteoarthritis cartilage degeneration by releasing histone deacetylase 4 inhibition of runt-related transcription factor-2 and increasing osteoarthritis-related genes: a novel mechanism of human osteoarthritis cartilage degeneration
Source: Arthritis Res Ther. 2014 Nov 26;16(6):491. doi: 10.1186/s13075-014-0491-3 (PMC4265470; doi:10.1186/s13075-014-0491-3)

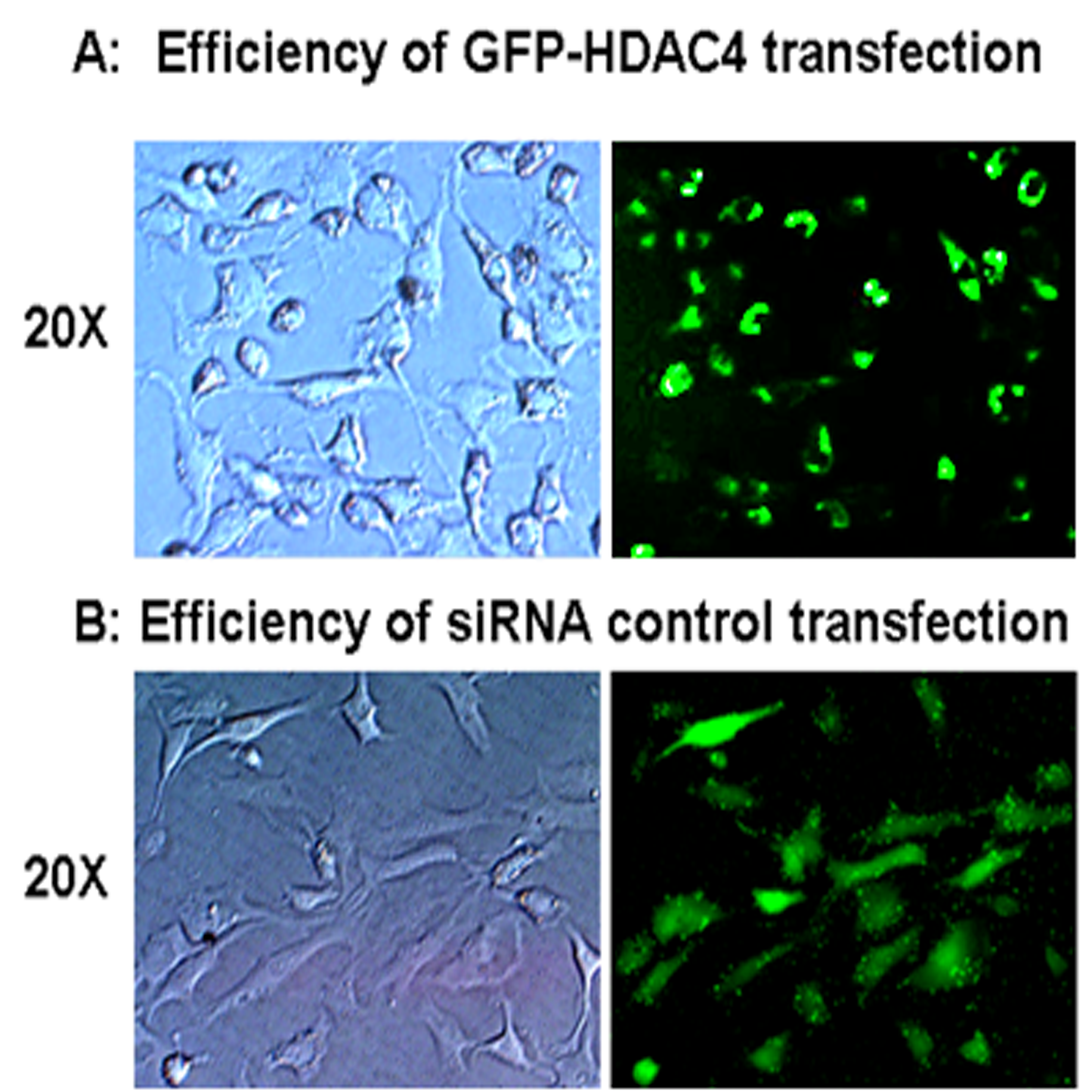

Supplement: Additional file 1: Figure S1. — The transfection efficiency of GFP-HDAC4 or siRNA control. (A) Efficiency of GFP-HDAC4 transfection was 79.1% (72.6% to 87.6%). (B) Efficiency of siRNA control transfection was 88.2% (85.4% to 91.2%). The percentage of cells that are GFP-positive was detected using fluorescence microscopy. Approximately 300 cells from three independent experiments were scored. [file 13075_2014_491_MOESM1_ESM.tiff]
